# Evaluation of the Low-Level Laser Therapy in Pain, Bite Force, and Mouth Opening Following Midfacial Trauma

**DOI:** 10.3390/life14121626

**Published:** 2024-12-09

**Authors:** Mateus Diego Pavelski, Maicon Douglas Pavelski, Luana Ferreira Oliveira, Helio Doyle Pereira da Silva, Lucio Frigo, Osvaldo Magro-Filho

**Affiliations:** 1 School of Dentistry, São Paulo State University, Araçatuba 16015-050, Brazil; maicon_pavelski@hotmail.com (M.D.P.); luana-ferreira.oliveira@unesp.br (L.F.O.); 2 Guarulhos University, Guarulhos 07023-070, Brazil; helio.silva@prof.ung.br (H.D.P.d.S.); luciofrigo@uol.com.br (L.F.)

**Keywords:** low-level light therapy, maxillofacial injuries, pain management, zygomatic fractures

## Abstract

Low-level laser therapy (LLLT) is known for its biostimulant properties, which can reduce inflammation and promote tissue regeneration. The present study is randomized, blinded, and placebo-controlled and aims to investigate the role of LLLT in the postoperative recovery of facial fractures. Patients with fractures of the zygomatic bone are selected and divided into two groups: low-level laser and red placebo light. The patients are evaluated for bite force, pain, mouth opening, and paresthesia in the pre-operatory day, on days 1, 2, 7, and 14. The trismus data demonstrated better results in the laser with a significant difference in the periods 7 and 14 days, with *p*-values of 0.0442 and 0.026, respectively. Regarding the bite force analyzed, no statistically significant differences were observed. In the pain scale comparison, there was a difference between the PLACEBO group and the LASER group for day 1 (*p* = 0.011), day 2 (*p* = 0.001), 7 (*p* = 0.001), and 14 days (*p* = 0.010). In the evaluation of paresthesia, on days 7 and 14, there were better results in the LASER group with *p* = 0.012 and *p* = 0.001. The laser acted as a moderator of the healing process, having a considerable effect on the aspects of trismus, pain, postoperative paresthesia, and bite force.

## 1. Introduction

The low-level laser therapy (LLLT) is known for its biostimulant properties, which can reduce inflammation and promote tissue regeneration. Studies suggest that LLLT may improve bone formation, angiogenesis, and collagen deposition. Additionally, LLLT may modulate the inflammatory response, promoting a more favorable environment for healing [1,2,3,4]. While the exact mechanism of action is still unclear, it is hypothesized that LLLT modulates inflammatory regulators and tissue repair, decreases nerve conduction, and antagonizes the effects of COX and cytokines [3,5].

The efficacy of LLLT in accelerating bone healing has been related to several preclinical and clinical studies. For example, Khadra et al. presented in a study with rats that LLLT significantly improved bone healing, as assessed by radiological and biomechanical analyses, while Dörtbudak et al. investigated the influence of LLLT on the osseointegration of dental implants, observing faster and more stable integration in patients treated with LLLT [4,6]. These findings may be relevant to treating facial fractures, where bone stability is crucial for recovery. From a clinical point of view, the LLLT offers several practical advantages [7,8].

Because it is a non-invasive modality, LLLT can be applied directly to the fracture area without additional surgical interventions, which is particularly beneficial in patients with comorbidities that may delay healing, suffer from drug interactions, or increase surgical risk. The study by Pretel et al. (2007) reported that LLLT facilitated bone repair in defects fixed with resorbable screws, indicating that LLLT can broadly act on fractured bone areas [1,8].

The literature suggests that LLLT can alleviate postoperative pain and edema, providing additional comfort during postoperative recovery. In addition to the biological effects of LT, its practical application has proven advantageous due to its non-invasive nature, absence of significant side effects, and ease of use [5,9,10].

The zygomatic bone is one of most fractured bone in the face due to its projected profile [11]. This study aims to evaluate the efficacy of LLLT in a clinical setting, comparing the outcomes of patients with CZM fractures treated with LLLT and a red placebo light. The analysis will address variables such as pain, paresthesia, and mouth opening, providing a comprehensive view of the efficacy of LLLT in different patient subgroups. Furthermore, low-level laser therapy (LLLT) has established itself as a promising approach in the management of facial trauma, with studies demonstrating its efficacy in reducing pain, edema, and trismus, improving mouth opening, and decreasing post-traumatic complications [1,5,6,7,8]. Recent systematic reviews point to the need for standardized protocols to optimize clinical results, ensuring greater efficacy and reproducibility [3,8,9,10,12]. This thesis, therefore, proposes to investigate the role of LLLT with standardization of parameters compared with a placebo red light in the postoperative recovery of facial fractures.

## 2. Materials and Methods

### 2.1. The Study Design and Participants

The present research is a randomized, blinded, and placebo-controlled study that was carried out in the São Paulo State University between March 2022 and November 2022. It was approved by the ethics committee of Western Saint Catherine University under protocol number 38991420.0.0000.5367 and followed all ethical principles and good clinical practices during its execution. All participants agreed to participate in the study and signed an informed consent form after a presentation of the study model, objectives, and guidelines.

The power of the sample (1 − β = 0.97) was calculated based on ANOVA with repeated measures from two groups, four repetitions, the correlation between measurements of 0.75, sample size *n* = 23, and α of 5%. The patients were admitted to the emergency room of the hospital of Araçatuba. The inclusion and exclusion criteria are presented in the following sections.

### 2.2. Inclusion Criteria

- Patients aged 15 to 75 years old;
- Absence of temporomandibular disorders;
- Absence of disabling systemic diseases and neuromuscular disorders;
- Patients with fractures of the zygomatic bone.

### 2.3. Exclusion Criteria

- Patients who refused to participate in the study;
- Patients with more facial fractures than the zygomatic bone and nasal bones;
- Patients with critical injuries in the intensive care unit;
- Patients who presented any postoperative complications.

### 2.4. Study Design

**Figure 1:**
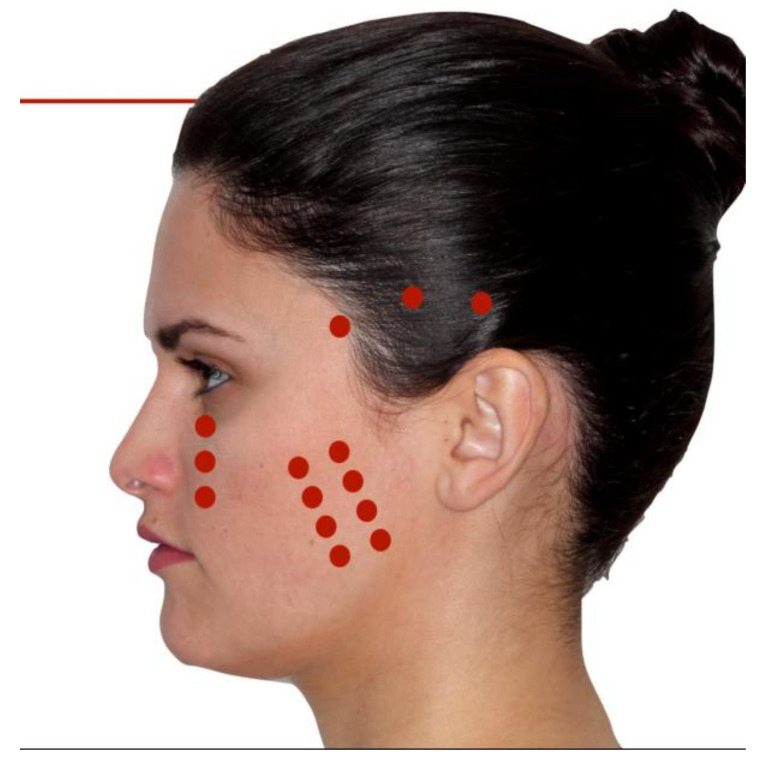
The red points indicate the irradiated area.

After clinical examination, tomographic analysis, and inclusion and exclusion criteria, 23 patients were selected to participate in this study. They were randomly assigned into two groups, named the “LASER group” (LG, *n* = 13) and the “PLACEBO group” (PG, *n* = 10). The randomization of the patient group was achieved by sorting the patients using two envelopes. Before surgery, each patient chose an envelope, and this defined which group the patient would belong to. There was no classifications regarding the fracture or the number of fixation points in the surgery. Both groups were irradiated at standardized points extra-orally by only one professional, distributed as eight points along the masseter muscle, three points on the temporalis muscle, and three points in the infraorbital nerve (Figure 1). In total, considering both sides of the face, 28 points were irradiated in each patient. Applications were performed in the immediate pre- and postoperative period at two, seven, and fourteen days after the surgery.

**Figure 2:**
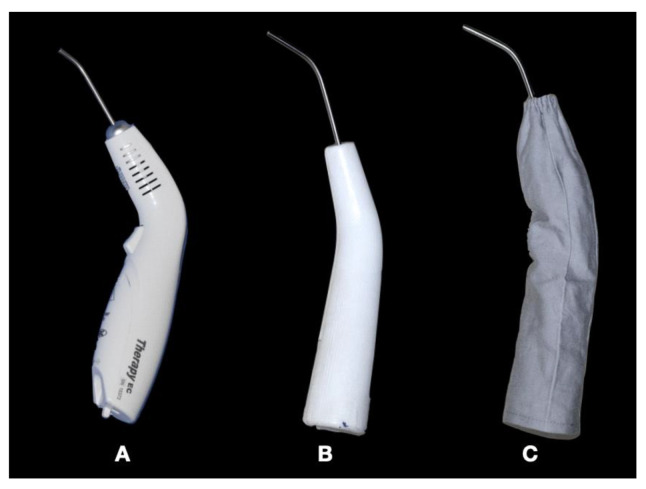
(**A**) Low-intensity laser used in the study. (**B**) Light emitter that mimics laser device. (**C**) Light emitter covered by the protective cover.

Photobiomodulation was performed in the LASER group using an aluminum gallium arsenate (AsGaAl) diode laser at a wavelength of 808 ± 10 nm (infrared light). Each point received 02 J (joules) of energy, a total of 44 J per session. The PLACEBO group received placebo treatment with a light generator produced to mimic a low-intensity laser device; however, its wavelength did not have any therapeutic effects. The two sources of light (laser and placebo) were covered with a protective cap, and thus the patient could not determine which treatment they were receiving (either the PBM or just the placebo light) as showed in Figure 2.

The clinical protocol of all surgeries was performed by specialized dental surgeons. The procedures were carried out by maintaining an aseptic chain and following the recommended technique following the AO recommendations [11]. All surgeries were performed under general anesthesia, with a nasal endotracheal tube, and the medications were standardized during the hospitalization time. During application, the patient’s face was clean and dry. Both the professional and the patient wore protective goggles during the PBM application procedure.

**Figure 3:**
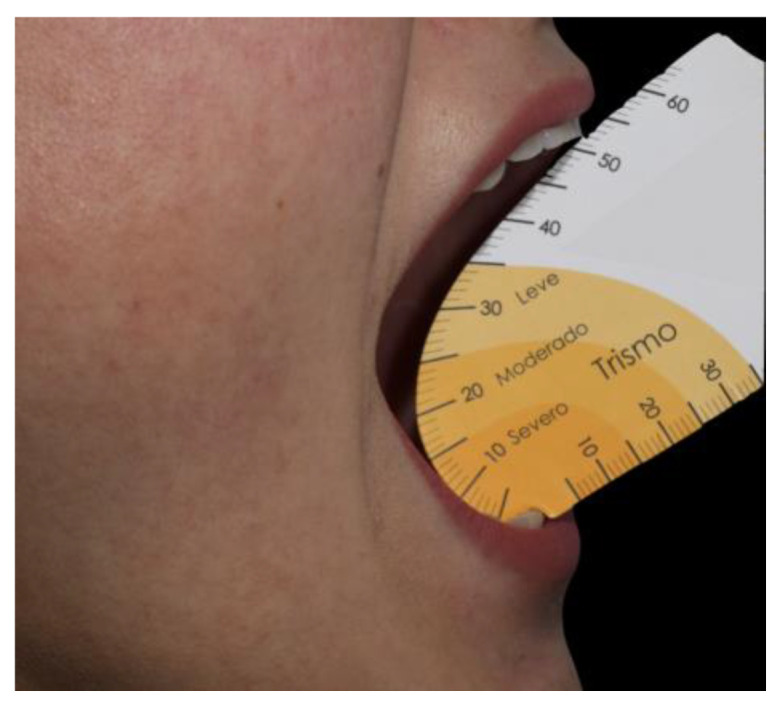
Ruler measuring the range of mouth opening.

Each patient was evaluated for mouth opening, bite force, touch sensibility, and pain in the first period, immediately post operation time and, two and, nine and, fourteen days post operation. The range of mouth opening was measured using a specific millimeter ruler. The ruler was positioned vertically in the interincisal space between the upper and lower central right incisors, and the patient was encouraged to open their mouth as wide as possible without pain, and this measure was defined as the open mouth distance (Figure 3).

The different degrees of trismus were divided into three categories, with a regular mouth opening defined as greater than 35 mm. Mild trismus was attributed to patients who had an opening between 30 and 35 mm. Individuals with mouth openings between 15 and 30 mm were classified as having moderate trismus, and patients with an opening smaller than 15 mm were classified as having severe trismus.

**Figure 4:**
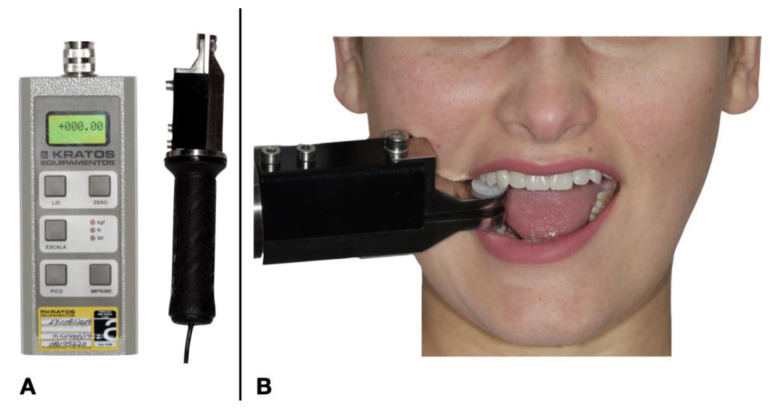
(**A**) Equipment used to measure bite force. (**B**) The equipment positioned at the measuring time.

Bite force was acquired with a gnathodynamometer and was measured in kilograms (Kratos Industrial Equipment Ltd., Cotia, SP, Brazil—Calibration Certificate R54408/19). The device load cell was positioned between the premolars on the opposite side of the fracture and the patient was instructed to bite the load cell to a limit that would not generate pain (Figure 4).

**Figure 5:**
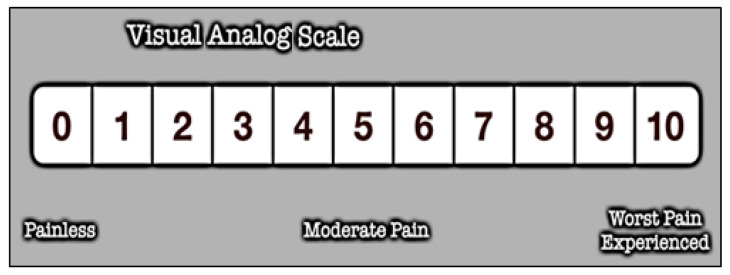
Visual analog pain scale used in the research.

The pain evaluation was carried out using a visual analog scale (VAS) that ranged from ten to zero (Figure 5), where ten was the worst pain experienced until zero, the total absence of pain by the patient. The visual analog scale was also used to assess paresthesia, with 0 being total paresthesia in the area and 10 being total sensitivity in the area.

Once the groups (PLACEBO/LASER) were delineated, the quantitative parameters of the study were summarized using the mean, standard deviation, median, and interquartile range. For qualitative parameters, absolute frequencies (*n*) and their respective percentages (%) were presented, and group comparisons were made using the chi-square test or Fisher’s exact test. To evaluate whether there was a difference in the means of quantitative parameters over time, a repeated measures ANOVA test was used, with the appropriate assumption checks (normality, independence, homogeneity of variances, and residual analysis) and post hoc adjustments with Bonferroni or Holm corrections, as applicable. The Mann–Whitney U test was applied to verify the homogeneity of the age and time to surgery parameters in both groups. A significance level of 5% was set in all analyses, and the data were analyzed using Jamovi^®^ (version 1.6) and R Core Team^®^ software (version 4.1.0).

## 3. Results

In the present study, almost 70% of the patients were men, with an average age of 33.1 years. In addition, 20 of the 23 patients had single fractures of the unilateral zygomatic orbital complex, while the 3 other patients had nasal fractures concomitant with zygomatic fracture. Most of the fractures were on the left side (65%), and most of the traumas were caused by motorcycle accidents, followed by physical aggression.

**Figure 6:**
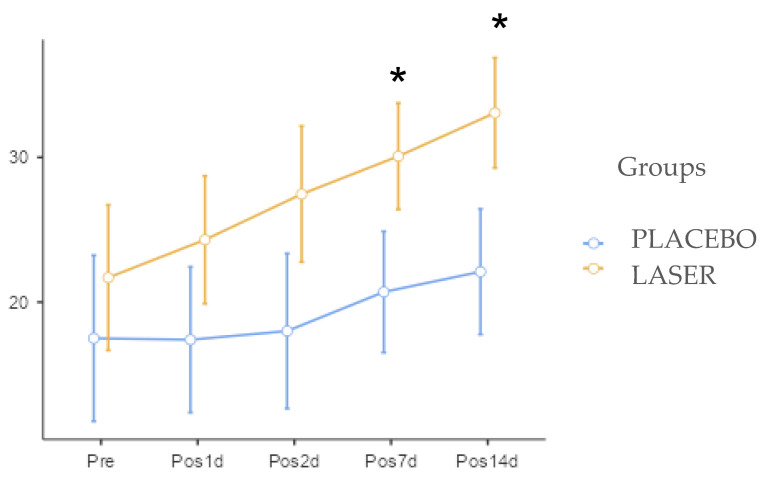
Mouth opening graph. The * indicate difference statistically significant.

The trismus data demonstrated better results in the LASER group in all detailed periods, as shown in Figure 6, but there was a significant difference only in the periods of 7 and 14 days, with *p*-values of 0.0442 and 0.026, respectively.

**Figure 7:**
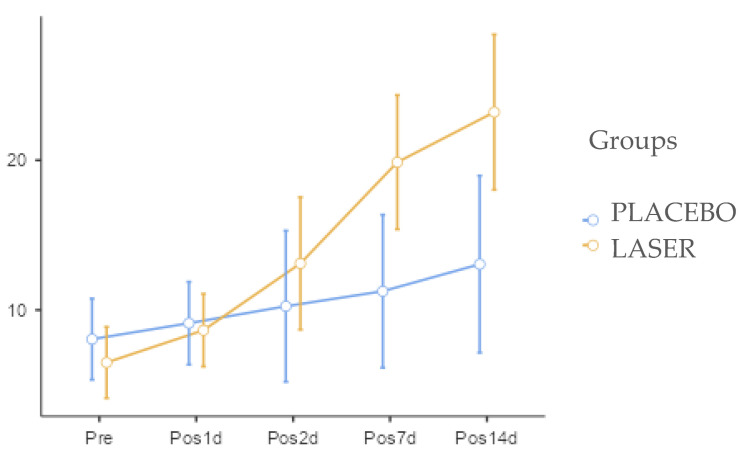
Bite force of the two groups. None difference statistically significant founded in this group.

Regarding the bite force analyzed, there were no statistically significant differences, despite slightly better results in the LASER group at two days until the end of the analysis as shown on Figure 7.

**Figure 8:**
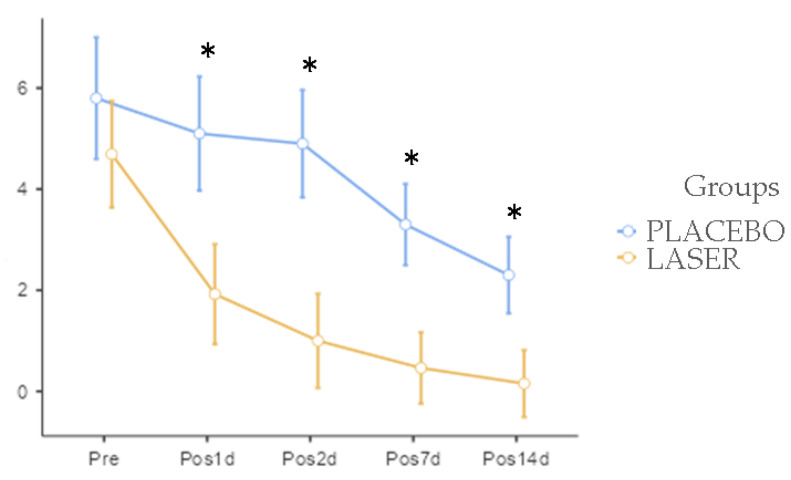
Graph of comparative visual analog scale of pain. There is lower scores of pain after surgery in LASER group, with statistics differences marked with “*”.

In the pain scale comparison, the Figure 8 demonstrate that there was a difference between the PLACEBO group and the LASER group at one day (*p* = 0.011), two days (*p* = 0.001), seven days (*p* = 0.001), and 14 days (*p* = 0.010) post operation.

**Figure 9:**
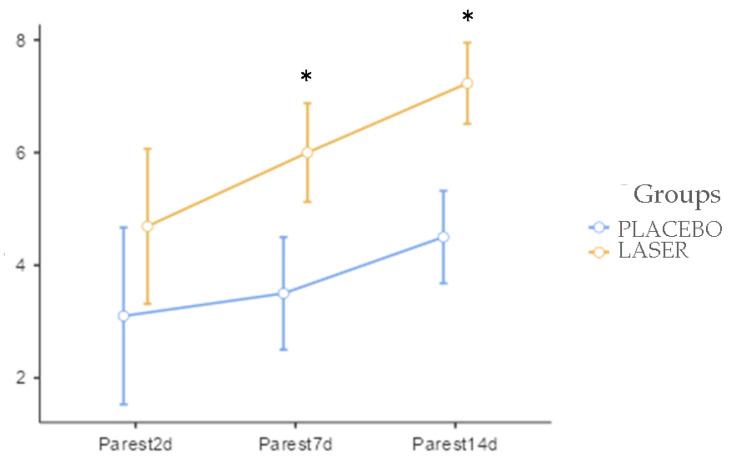
Evaluation of post-operatory sensibility perception, the periods with “*” marked are the periods with significance level above 95%.

Paresthesia was not analyzed in the immediate postoperative period. It was evaluated on the following day. The Figure 9 demonstrates that on day 2, there were no significant differences between the groups. On days 7 and 14, there were significantly better results in the LASER groups at *p* = 0.012 and *p* = 0.001, respectively.

## 4. Discussion

The benefits of using low-intensity lasers are well-described in the literature; their practicality, lack of adverse effects, and non-invasive nature contribute to a wide range of recommendations. In surgical recovery specifically, improvement in pain has already been mentioned [7,8,9,13], as has their benefits regarding trismus, paresthesia [2], and bite force [8], such as in third molars, implants, and facial traumas [2,10,13].

Firstly, the profile of facial traumas presented in this study is in agreement with the literature, being mostly men and young adults, with the etiological agents being traffic accidents and interpersonal violence.

Second, regarding trismus, the present study is in line with other studies in that it did not observe a statistical difference at the initial moment, but then saw a significant improvement after laser sessions, especially after two weeks [2,5,9]. Another study shows improvement after 30 days [8]. While only one study showed no significant improvement with laser treatment in comparison with placebo [7], there was a direct correlation with the number of weekly laser therapy sessions associated with a latent recovery time of two weeks after trauma. The only study that did not show improvements in mouth opening performed only one weekly laser application session, and all the others performed at least two weekly sessions.

In the analysis of bite force, the present study showed slightly better results in the LASER group, but without statistical difference. Corroborating the literature, the only study that demonstrated a difference reported significant results only after 60 days of trauma [8]. This could be explained by the short period analyzed for this sample.

Regarding pain results, this study demonstrated significant differences in all periods analyzed, at one, two, seven, and fourteen days. This coincides with previously published studies, with the only difference among these studies being the time of statistical significance. In Bashiri’s study [14], the PBM group showed better results in all four weeks analyzed, with a significant difference after one month of treatment [7]. This corroborates the study by Lauriti [8], which did not show any statistical differences until the thirtieth day. Other studies demonstrate a much better response time, with improvements at two weeks [9] and on the fourth day [5] (the latter being a systematic review). In a similar study that evaluated CZM fractures, a difference in the pain scale was also found [5]; however, this was only evident at one month post operation, which can be explained due to the shorter sessions of 5 s in each instance.

The sensitivity of the facial region is a question for which laser treatment has significant evidence since studies in animals have demonstrated its effectiveness after neurotmesis in rats at 1.5 months with at least two sessions per week [2,15]. The present study demonstrates a subtle improvement after two days and a statistically significant improvement after 7 (*p* = 0.012) and 14 days (*p* = 0.001). In comparison to the only published study evaluating zygomatic fractures, an improvement in sensitivity was reported; however, this was evident only after 1 month of laser treatment with two weekly sessions [14]. Most studies published on paresthesia in facial trauma are on mandibular fractures, analyzing the inferior alveolar nerve and presenting contrasting results. Some authors reported no differences [7], while others resulted in significant improvements from 7 days to 6 months with tactile, thermal, and two-point differences [16]. A systematic review and meta-analysis of mandible fractures concluded that PBM has no significant effects in short periods of up to two weeks, but is more substantiated after 30 days [10]. This difference could be explained by the larger diameter of the inferior alveolar nerve and the mobility of the fractured jaw until surgery, which could worsen the damage.

## 5. Conclusions

In this study, the laser acted as an accelerator of the healing process, having a considerable effect on the aspects of trismus, pain, and postoperative paresthesia and less significantly on bite force, where there was a slight improvement. We can determine that the laser improves this postoperative period, reducing pain, trismus, and paresthesia, allowing the patient less morbidity and a better quality of life, and is thus an excellent tool for non-pharmacological support. In addition, we need larger long-term studies comparing laser protocols to standardize the criteria for low-intensity laser therapy.

## Data Availability

The data presented in this study are available on request from the corresponding author.
